# Multi-Institutional Survey of Fourth Year Students’ Self Assessed Milestone Based Skill Proficiency and Faculty Expectation During an Emergency Medicine Clerk-ship: Implications For Curriculum Development

**DOI:** 10.15694/mep.2018.0000212.1

**Published:** 2018-09-13

**Authors:** Katie Pettit, Joseph Turner, Kathryn Hogan, Stacey Poznanski, Camiron Pfennig-Bass, Sarkis Kouyoumjian, Braden Hexom, Anthony Perkins, Cory Pitre, Aloysius Humbert

**Affiliations:** 1 Indiana University School of Medicine; 2 Albany Medical College; 3 Wright State University; 4 Vanderbilt University (now affiliated with University of South Carolina School of Medicine Greenville); 5 Wayne State University; 6 Icahn School of Medicine at Mt. Sinai; 7 Indiana University Center for Health Innovation and Implementation Science

**Keywords:** medical students, faculty expectations, milestones, proficiency

## Abstract

This article was migrated. The article was marked as recommended.

**Introduction:** Emergency medicine milestones suggest skill performance expectations for graduating medical students. The objective of this study is to examine differences between student’s perceived proficiency and faculty expectations relative to Level 1 EM milestones, identifying opportunities for curriculum development.

**Methods:** Using ACGME milestone language, the authors developed a survey that measures student perceived skill proficiency with 22 skills, which was administered to fourth year medical students at 6 institutions. Similar surveys were sent to faculty to determine their expectations of students’ skill proficiency. Differences between student and faculty responses were calculated.

**Results:** There were 608 student and 114 faculty responses. There was a statistically significant difference between mean student and faculty responses for 13 of the 22 skills. For 10 of these skills, students rated their own skill proficiency higher than faculty expectations. For 3 of the skills, faculty rated their expectations higher than students’ perceived proficiency.

**Conclusions:** For pharmacology skills, student ratings were low, indicating an area to focus curriculum development. Items where student ratings are higher than faculty may be a result of overconfidence or a lack of understanding by faculty of students’ abilities. Formal assessment of skills in these areas would help clarify the reason and direct faculty and curriculum development.

## Introduction

The Accreditation Council for Graduate Medical Education (ACGME) and the American Board of Emergency Medicine (ABEM) released the Emergency Medicine (EM) Milestones in 2012 ( Beeson et al., 2013). Incoming interns are expected to have achieved a Level 1 on each EM Milestone prior to medical school graduation.

Although the Level 1 Milestones are expected of an incoming intern, these objectives are not uniformly used for teaching and assessing students during their undergraduate medical education ( Santen et al., 2013). In 2014, the AAMC released the Core Entrustable Professional Activities for Entering Residency (CEPAERs), a common set of core behaviors that should be expected of all graduates. While the CEPAERs provide guidance, medical schools still maintain the flexibility to set their own educational objectives and graduation requirements ( Colleges, 2015). This variability in clinical performance expectations does not ensure that Level 1 Milestones are consistently met prior to graduation, and this sets the stage for varying skill levels among medical students and interns, as well as varying expectations among faculty and program directors.

Incongruent expectations of clinical abilities between faculty and learners have been demonstrated across different specialties ( Dickson et al., 2013; Wenrich et al. 2010) as well as outside of United States training programs ( Sicaja et al., 2006). The literature has also demonstrated differing expectations in regards to the learner’s involvement in patient care ( Quillen et al., 2013) and procedural skills ( Dickson et al., 2013). These varied expectations potentially yield inconsistent student supervision leading to possible patient safety implications.

Unique characteristics common to most EM clerkships introduce even more inconsistency in expectations. The nature of EM scheduling results in medical students interacting with many different faculty during the clerkship - often during any one shift. When layering the increased autonomy that is experienced on many EM clerkships with inconsistent faculty expectations, students are disadvantaged without clear faculty expectations.

Given that the EM Milestones are an expected starting point for incoming EM interns, the authors assessed student self-perceived skill proficiency and comfort as well as faculty expectations of EM milestone based skill proficiency. Our objective was to measure the differences between faculty expectation and student confidence with respect to student performance of Milestone-derived behaviors.

## Methods

We conducted a prospective observational study at six academic institutions throughout the United States. The study was approved by the Institutional Review Boards of the participating institutions. These institutions were a convenience sampling based on participation interest through the Clerkship Directors Emergency Medicine listserv. All institutions had a required emergency medicine clerkship during the time of the study. Four of the institutions identify themselves as university affiliated, and four identify as an urban institution.

Fourth-year medical students enrolled in a 4-week emergency medicine course were approached for enrollment and provided written consent during emergency medicine clerkship orientation. EM faculty at all 6 institutions were provided a study information sheet, and faculty participation in an online survey served as consent.

The survey instrument was designed by the authors and contained language taken from Level 1 EM Milestones (see Figure 1S in the supplementary files). It contained 22 items with each item being rated on a 5-point Likert scale. Fifteen of these items asked students to rate their skill proficiency, and 7 of these items asked the students to rate their comfort in performing a skill. Students completed the survey at the beginning of the clerkship and a second time after completing the clerkship.

Faculty were randomized and received electronic surveys at one of three separate times during the study; July, November or March. These time frames were chosen to represent expectations of students in the early, middle, and later part of the year. The faculty survey was very similar to the medical student survey (see Figure 2S in the supplementary files) differing only in language to reflect expectations rather than self-assessed proficiency or comfort. Faculty were instructed to complete the survey based on expectations for fourth year students at the beginning of their EM clerkship at the time of year that the survey was completed.

Data from the faculty surveys were entered automatically into REDCap, an electronic data capture tool hosted at the home institution ( Harris et al., 2009). REDCap (Research Electronic Data Capture) is a secure, web-based application designed to support data capture for research studies, providing: 1) an intuitive interface for validated data entry; 2) audit trails for tracking data manipulation and export procedures; 3) automated export procedures for seamless data downloads to common statistical packages; and 4) procedures for importing data from external sources. A Masters of Public Health student was responsible for obtaining medical student consent, administering the surveys, and entering this data into REDCap. From this point, data was only viewed as aggregate and individual student data could not be identified.

### Data Analysis

All item comparisons between faculty and student responses were tested using Wilcoxon-Rank Sum tests since the items were measured on an ordinal Likert scale. For each set of comparisons, p-values were adjusted using the Stepdown Bonferroni method. Since the surveys were collected at different times of the year, we examined the effect of survey time collection on faculty responses and student’s pre-clerkship responses. Specifically, we tested whether the difference between the two sets of responses differed by testing the interaction between time period and response type (student pre-clerkship vs. faculty) in a proportional odds model for each item. We did not have identifiers that enabled linking of a student’s pre-clerkship and post-clerkship response. Therefore, we were not able to perform a paired analysis, and used the Wilcoxon-Rank Sum test for all item comparisons. We performed analyses using the dichotomy of 4/5 versus all other scores. Results were similar, therefore we only report results using mean scores. All analyses were performed using SAS V9.3.

## Results/Analysis

Surveys were returned by 114 faculty: 33 during the early time period (July), 57 during the middle time period (November), and 24 during the late time period (March). A total of 426 faculty were sent surveys across all six participating sites with a total response rate of 27%. A total of 608 students returned pre-clerkship surveys and 533 students returned post-clerkship surveys. Surveys were given to 908 students with a pre-clerkship response rate of 67% and a post-clerkship response rate of 59%.

Differences in students’ self-assessed proficiency at the beginning of the EM clerkship compared with faculty expectations as a mean score can be seen in Table 1.

**Table 1.** Mean student comfort pre-clerkship compared with mean faculty expectations. Light grey shading indicates higher student comfort; dark grey shading indicates higher faculty expectations.

	Faculty			Pre-Clerkship			P-value ^*^
	N	Mean	Std. Dev.	N	Mean	Std. Dev.	
Comprehensive History & Physical Exam	114	4.04	0.70	606	3.94	0.61	*0.702*
Listening Effectively	113	4.33	0.67	607	4.39	0.63	*1.000*
Eliciting Patient Expectations	113	3.89	0.79	606	4.18	0.62	***0.004***
Constructing a List of Diagnoses	114	3.59	0.73	608	3.45	0.67	*0.644*
Recognize abnormal vital signs	112	3.76	0.81	605	3.69	0.77	*1.000*
Necessity/Urgency of Diagnostic Studies	113	3.12	0.72	606	3.27	0.76	*0.336*
Planning Therapeutic Interventions	113	3.00	0.74	604	3.23	0.69	***0.021***
Ordering Medications Correctly	112	2.73	1.04	607	2.10	0.87	***0.002***
Pharmacologic Knowledge	113	3.33	0.87	608	2.86	0.93	***0.002***
Asking about Allergies	114	4.27	0.92	606	3.93	0.97	***0.002***
Performing Procedures	113	2.81	0.93	605	3.20	1.06	***0.003***
Understanding Available Resources	113	2.54	0.95	607	3.28	0.92	***0.002***
Multi-tasking	113	3.29	0.94	608	3.56	0.83	***0.037***
Using Ultrasound	113	2.47	0.97	608	3.13	0.90	***0.002***
Establishing Rapport with Patients	113	4.05	0.64	607	4.41	0.63	***0.002***
Demonstrating Empathy	112	4.38	0.63	597	4.47	0.66	*0.702*
Demonstrating Caring Behavior	113	4.35	0.61	600	4.50	0.62	***0.081***
Participate in Peer Teaching	112	2.81	1.07	599	3.75	0.80	***0.002***
Navigating Health Care System	114	2.25	0.92	594	3.15	0.84	***0.002***
Using Electronic Health Record	113	3.05	1.07	600	3.87	0.82	***0.002***
Computer Physician Order Entry	112	2.70	1.19	598	2.60	1.18	*1.000*
Participating as part of a Team	113	3.71	0.80	598	3.85	0.80	*0.702*

^*^ P-value has been adjusted using the Stepdown Bonferroni Method

There was a statistically significant difference for 13 of the 22 skills. For 10 of the statistically significant skills, students rated their own proficiency higher than faculty expectations: eliciting patient expectations, planning therapeutic interventions, performing procedures, understanding available resources, multi-tasking, using ultrasound, establishing rapport with patients, participating in peer teaching, navigating the healthcare system, and using the electronic health record (EHR). For 3 of the statistically significant skills, faculty rated their expectations higher than student’s proficiency: ordering medications correctly, pharmacologic knowledge, and asking about allergies.

Changes in student responses between pre-clerkship and post-clerkship surveys can be seen in Table 2.

**Table 2.** Difference between student comfort pre and post-clerkship.

	Pre-Clerkship			Post-Clerkship			P-value ^*^
	N	Mean	Std Dev.	N	Mean	Std Dev.	
Comprehensive History & Physical Exam	606	3.94	0.61	532	4.30	0.53	0.002
Listening Effectively	607	4.39	0.63	533	4.59	0.52	0.002
Eliciting Patient Expectations	606	4.18	0.62	530	4.42	0.57	0.002
Constructing a List of Diagnoses	608	3.45	0.67	530	4.01	0.57	0.002
Recognize abnormal vital signs	605	3.69	0.77	532	4.23	0.65	0.002
Necessity/Urgency of Diagnostic Studies	606	3.27	0.76	530	3.95	0.65	0.002
Planning Therapeutic Interventions	604	3.23	0.69	531	3.83	0.62	0.002
Ordering Medications Correctly	607	2.10	0.87	533	2.90	0.96	0.002
Pharmacologic Knowledge	608	2.86	0.93	530	3.38	0.92	0.002
Asking about Allergies	606	3.93	0.97	533	4.33	0.84	0.002
Performing Procedures	605	3.20	1.06	532	4.00	0.84	0.002
Understanding Available Resources	607	3.28	0.92	531	3.77	0.83	0.002
Multi-tasking	608	3.56	0.83	531	4.11	0.69	0.002
Using Ultrasound	608	3.13	0.90	531	3.83	0.78	0.002
Establishing Rapport with Patients	607	4.41	0.63	533	4.66	0.52	0.002
Demonstrating Empathy	597	4.47	0.66	529	4.62	0.56	0.002
Demonstrating Caring Behavior	600	4.50	0.62	527	4.66	0.53	0.002
Participate in Peer Teaching	599	3.75	0.80	527	4.08	0.72	0.002
Navigating Health Care System	594	3.15	0.84	530	3.72	0.82	0.002
Using Electronic Health Record	600	3.87	0.82	525	4.17	0.75	0.002
Computer Physician Order Entry	598	2.60	1.18	526	3.35	1.15	0.002
Participating as part of a Team	598	3.85	0.80	530	4.28	0.66	0.002

^*^ P-value has been adjusted using the Stepdown Bonferroni Method

For all 22 skills there was a statistically significant improvement in student comfort from the beginning to the end of the clerkship.

Table 3 presents student self-assessed proficiency at the conclusion of the EM clerkship compared with faculty expectations as a mean score.

**Table 3.** Faculty expectations compared with post-clerkship student comfort. Shading indicates higher student comfort.

	Faculty			Post			P-Value ^*^
	N	Mean	Std. Dev.	N	Mean	Std. Dev.	
Comprehensive History & Physical Exam	114	4.04	0.70	532	4.30	0.53	**0.002**
Listening Effectively	113	4.33	0.67	533	4.59	0.52	**0.002**
Eliciting Patient Expectations	113	3.89	0.79	530	4.42	0.57	**0.002**
Constructing a List of Diagnoses	114	3.59	0.73	530	4.01	0.57	**0.002**
Recognize abnormal vital signs	112	3.76	0.81	532	4.23	0.65	**0.002**
Necessity/Urgency of Diagnostic Studies	113	3.12	0.72	530	3.95	0.65	**0.002**
Planning Therapeutic Interventions	113	3.00	0.74	531	3.83	0.62	**0.002**
Ordering Medications Correctly	112	2.73	1.04	533	2.90	0.96	0.207
Pharmacologic Knowledge	113	3.33	0.87	530	3.38	0.92	1.000
Asking about Allergies	114	4.27	0.92	533	4.33	0.84	1.000
Performing Procedures	113	2.81	0.93	532	4.00	0.84	**0.002**
Understanding Available Resources	113	2.54	0.95	531	3.77	0.83	**0.002**
Multi-tasking	113	3.29	0.94	531	4.11	0.69	**0.002**
Using Ultrasound	113	2.47	0.97	531	3.83	0.78	**0.002**
Establishing Rapport with Patients	113	4.05	0.64	533	4.66	0.52	**0.002**
Demonstrating Empathy	112	4.38	0.63	529	4.62	0.56	**0.002**
Demonstrating Caring Behavior	113	4.35	0.61	527	4.66	0.53	**0.002**
Participate in Peer Teaching	112	2.81	1.07	527	4.08	0.72	**0.002**
Navigating Health Care System	114	2.25	0.92	530	3.72	0.82	**0.002**
Using Electronic Health Record	113	3.05	1.07	525	4.17	0.75	**0.002**
Computer Physician Order Entry	112	2.70	1.19	526	3.35	1.15	**0.002**
Participating as part of a Team	113	3.71	0.80	530	4.28	0.66	**0.002**

* P-value has been adjusted using the Stepdown Bonferroni Method

For 19 skills, students rated their comfort significantly higher than faculty expectations. For 3 skills (ordering medications correctly, pharmacologic knowledge, and asking about allergies) there was no significant difference. At the beginning of the clerkship, students rated their skill proficiency in these areas significantly lower than faculty, but this difference disappeared by the end of the clerkship. This suggests that students’ skill proficiency in the area of pharmacology is improved over the course of the clerkship.

Data comparing student self-assessed skill proficiency at the beginning of the EM clerkship with faculty expectations divided by the early, middle, and late academic year can be found in Tables 4- 6.

**Table 4.** Pre-clerkship student comfort compared to early academic year faculty expectations.

	Faculty			Pre-Clerkship		
	N	Mean	Std. Dev	N	Mean	Std. Dev.
H_P	33	3.85	0.76	261	3.88	0.57
Listening_Effectively	33	4.24	0.66	261	4.36	0.61
Eliciting_Info	33	4.00	0.66	262	4.15	0.58
Diagnoses	33	3.58	0.56	262	3.42	0.64
Vitals_ABCs	32	3.59	0.80	261	3.67	0.76
Diagnostic_Studies	33	2.94	0.70	262	3.22	0.76
Interventions	33	2.88	0.70	261	3.20	0.67
Medications	33	2.67	1.02	262	2.02	0.82
Pharm_Mech	33	3.18	0.77	262	2.90	0.89
Allergies	33	4.33	0.82	260	3.87	1.01
Procedures	32	2.78	0.97	259	3.18	1.00
Resources	33	2.58	0.94	261	3.21	0.87
Multi_task	33	3.24	0.97	262	3.57	0.80
Ultrasound	32	2.34	0.83	262	3.06	0.87
Rapport	32	4.06	0.56	262	4.35	0.63
Empathy	33	4.30	0.53	258	4.45	0.64
Caring	32	4.41	0.50	258	4.48	0.64
Teach	32	2.63	0.91	258	3.69	0.81
System	33	2.21	0.89	253	3.09	0.85
E_H_R_	32	3.09	0.96	258	3.75	0.85
CPOE	32	2.84	1.30	257	2.36	1.06
Team	33	3.70	0.53	258	3.78	0.81

**Table 5.** Pre-clerkship student comfort compared to mid-academic year faculty expectations.

	Faculty			Pre-Clerkship		
	N	Mean	Std. Dev	N	Mean	Std. Dev
H_P	57	4.09	0.61	161	4.00	0.59
Listening_Effectively	56	4.38	0.65	161	4.46	0.61
Eliciting_Info	56	3.93	0.85	160	4.24	0.62
Diagnoses	57	3.60	0.82	161	3.45	0.70
Vitals_ABCs	57	3.81	0.81	160	3.65	0.79
Diagnostic_Studies	56	3.25	0.74	161	3.23	0.78
Interventions	56	3.11	0.80	160	3.19	0.73
Medications	55	2.85	1.80	161	2.12	0.88
Pharm_Mech	57	3.35	0.95	161	2.86	0.85
Allergies	57	4.30	0.98	161	3.98	0.96
Procedures	57	2.88	0.98	161	3.24	1.15
Resources	56	2.57	1.02	161	3.29	0.98
Multi_task	56	3.36	0.96	161	3.52	0.87
Ultrasound	57	2.60	1.07	161	3.06	0.91
Rapport	57	3.98	0.69	160	4.53	0.56
Empathy	55	4.44	0.66	157	4.56	0.63
Caring	57	4.37	0.64	159	4.59	0.55
Teach	57	3.02	1.16	158	3.82	0.84
System	57	2.35	1.01	158	3.21	0.88
E_H_R_	57	2.96	1.09	159	3.94	0.83
CPOE	56	2.64	1.17	158	2.69	1.20
Team	57	3.75	0.85	159	3.91	0.77

**Table 6.** Pre-clerkship student comfort compared to late academic year faculty expectations.

	Faculty			Pre-Clerkship		
	N	Mean	Std. Dev.	N	Mean	Std. Dev.
H_P	24	4.17	0.82	184	3.96	0.66
Listening_Effectively	24	4.33	0.76	185	4.37	0.67
Eliciting_Info	24	3.67	0.82	184	4.18	0.68
Diagnoses	24	3.58	0.72	185	3.50	0.68
Vitals_ABCs	23	3.87	0.81	184	3.76	0.75
Diagnostic_Studies	24	3.08	0.65	183	3.36	0.74
Interventions	24	2.92	0.65	183	3.31	0.68
Medications	24	2.54	0.98	184	2.21	0.93
Pharm_Mech	23	3.48	0.79	185	2.80	0.98
Allergies	24	4.13	0.95	185	3.96	1.01
Procedures	24	2.67	0.76	185	3.18	1.08
Resources	24	2.42	0.78	185	3.36	0.93
Multi_task	24	3.21	0.88	185	3.58	0.84
Ultrasound	24	2.33	0.92	185	3.27	0.91
Rapport	24	4.21	0.59	185	4.40	0.68
Empathy	24	4.38	0.71	182	4.40	0.69
Caring	24	4.25	0.68	183	4.44	0.65
Teach	23	2.57	0.99	183	3.78	0.76
System	24	2.04	0.69	183	3.17	0.80
E_H_R_	24	3.21	1.18	183	3.97	0.75
CPOE	24	2.63	1.13	183	2.86	1.25
Team	23	3.61	0.99	181	3.88	0.81

The interaction between time and response person revealed 3 items with marginally significant interactions (diagnostic studies, p = 0.09; teaching, p = 0.06; and CPOE, p = 0.08). While not significant, this indicates there may be differences between the way students and faculty respond throughout the year. Additionally, several variables showed that faculty and student pre-clerkship levels changed consistently over time. These variables were history and physical exam (p = 0.04), demonstrating empathy (p = 0.04), and using electronic health records (p = 0.02). Specifically, scores for both faculty and students were lowest in period 1 for history and physical exam, and using electronic health records, while the demonstrating empathy was highest in period 2. It is reasonable to assume that expectations and self-assessed skill proficiency would be lower at the beginning of students’ final year and higher towards the end. It is less clear why empathy would be highest in the middle of the academic year.

## Discussion

Our study provides a quantitative analysis regarding differences between student self-assessed skill proficiency and faculty expectations across a range of skills considered mandatory for incoming interns in EM programs. The expectation is that students going into EM are proficient in these skills by medical school graduation. Previous studies have revealed significant differences between student and faculty expectations of skills at the start of medical school clinical years ( Wenrich et al., 2010) and at the beginning of a family practice residency ( Dickson et al., 2013). While we examined learners at a different stage of training and assessed a different set of skills, our study confirmed that there is a significant difference between student self -assessed proficiency and faculty expectations.

In most areas, students beginning their EM clerkship rated their skill proficiency higher than faculty expectations. In particular, students reported increased skill proficiency related to systems-based care and technical proficiency. This raises the question of whether faculty underestimate the skill proficiency of incoming students or whether students overestimate their own skill proficiency. The former may represent an opportunity for faculty development. Aligning faculty expectations with actual student proficiencies could prevent redundancy in teaching and make more efficient use of instructional time. The latter possibility, that students overestimate their own skill proficiency, is more concerning as it could potentially compromise patient safety if students attempt to carry out tasks for which they are not proficient without seeking adequate faculty supervision.

Students in our study were significantly less comfortable with medications than faculty expected them to be. With the exception of entering orders in CPOE, which one might surmise would include medication orders, all of the skills in which students felt less proficient than faculty expectations had specifically to do with medications. This may represent a curricular gap in the preclinical and early clinical years. However, even the late-year comparison indicated students felt less proficient with pharmacologic knowledge than faculty expected. This raises the possibility that pharmacologic education may be a shortcoming throughout medical school and an important area for further curricular development.

### Limitations

There are some potential limitations to our study. We surveyed students at multiple institutions to provide a broader and more generalizable assessment than could be obtained from a single-center study, but this does not guarantee that the data are applicable to all institutions across the country. Furthermore, we did not record the institution for each returned survey so we cannot assess for differences from one site to another. Additionally, since we did not record individual student identifiers and students frequently do away rotation at multiple institutions, it is possible that a small number of students were administered the survey twice. Finally, not all senior medical students in this survey were applying for an Emergency Medicine residency. The EM Milestones were specifically designed to quantify skills for EM residents. Faculty may have different expectations of students based on their intended field of practice. Our study did not address this, and we did not collect information regarding students’ future training plans.

Some educators may suggest using tools other than the EM Milestones to generate a list of skills of interest. Recently, EM physicians have proposed an additional milestone list specifically targeted to medical students ( Santen et al., 2014). The American Association of Medical Colleges has also released a list of Entrustable Professional Activities that medical students are expected to demonstrate prior to advancing to residency training ( Colleges, 2015). At the time of our data collection neither of these lists had been published. Nevertheless, when developing our survey we did not use every action included in the Level 1 EM Milestones. Rather, we extracted skills that are broadly applicable across multiple specialties and which we believe represent a reasonable expected skill set for any new resident. More importantly, surveys generated from the EM student milestones or CEPAERs may have missed the pharmacologic concerns discovered in our study as both lists mention therapeutics, but neither specifically references medications.

Future work in this area should include assessment of student skill proficiency to determine whether actual student performance aligns more closely with student or faculty expectations. Additional research should also focus on our finding of higher faculty expectations in regards to pharmacologic education and the possible implication on patient safety.

## Conclusion

Across a range of core clinical skills, medical student self-assessed proficiency differs significantly from faculty expectations. Specifically, in the area of pharmacology, students feel significantly less proficient than faculty expect them to be, and this may be an area for focused curriculum development. For items where student self-assessed proficiency is significantly higher than faculty expectations, formal assessment of the student’s ability in these areas may be useful in clarifying whether this discrepancy is due to student overconfidence or a lack of faculty understanding of students’ abilities and in providing direction for both future curriculum development and faculty development.

## Take Home Messages

• There are significant differences between medical students’ perception of their skill level and faculty expectations for student skill level across a wide range of skills.
• Medical students rate themselves as having low proficiency in pharmacology
• Further research is required to clarify whether items where student self-assessed proficiency is significantly higher than faculty expectations are due to student overconfidence or a lack of faculty understanding of students’ abilities.

## Notes On Contributors

***Katie E. Pettit***, MD, is the Emergency Medicine Residency Associate Program Director at Indiana University, Indianapolis, Indiana.

***Joseph Turner***, MD, is the Emergency Medicine Residency Assistant Program Director and at the time of the study the Medical Student Clerkship Director at Indiana University, Indianapolis, Indiana.

***Kathryn Hogan***, MD, is the Medical Student Clerkship Director at Albany Medical College Department of Emergency Medicine, Albany, New York.

***Stacey Poznanski***, DO, is Emergency Medicine Residency Associate Program Director and Director of Undergraduate Medical Education at Wright State University, Dayton, Ohio.

***Camiron Pfennig-Bass***, MD, is the Emergency Medicine Residency Program Director at the University of South Carolina School of Medicine, Greenville, South Carolina, and formerly the Emergency Medicine Clerkship Director at Vanderbilt University, Nashville, Tennessee.

***Sarkis Kouyoumjian***, MD, is the Emergency Medicine Clerkship Director at Wayne State University, Detroit, Michigan.

***Braden Hexom***, MD, is the Emergency Medicine Residency Program Director at Rush University, Chicago Illinois, and formerly the Emergency Medicine Clerkship Director at Icahn School of Medicine at Mt. Sinai, New York, New York.

***Anthony Perkins***, MS, is a biostatistician with the Indiana Clinical and Translational Science Institute, Indianapolis, Indiana.

***Cory Pitre***, MD, is the Transitional Residency Program Director at IU Health Methodist Hospital, Indianapolis, Indiana.

***Aloysius Humbert***, MD, is the Emergency Medicine Residency Program Director and formerly Medical Student Clerkship Director at Indiana University, Indianapolis, Indiana.
