# Supplementary material for: Multi-Institutional Survey of Fourth Year Students’ Self Assessed Milestone Based Skill Proficiency and Faculty Expectation During an Emergency Medicine Clerk-ship: Implications For Curriculum Development
Source: MedEdPublish (2016). 2018 Sep 13;7:212. [Version 1] doi: 10.15694/mep.2018.0000212.1 (PMC10704418; doi:10.15694/mep.2018.0000212.1)
Supplement: Peer review history

# Reviewer report: Reviewer response for version 1

**DOI:** 10.21956/mep.19635.r29246

**Published:** 2018-09-19

**Authors:** Peter Dieter

**Affiliations:** 1 AMSE

This review has been migrated. The reviewer awarded 3 stars out of 5 The objective of the study was to identify differences between student´s perceived proficiency and faculty expectations related to Level EM milestones. Opportunities for curriculum development should be identified. 22 skills were used, 608 4th year medical students and 114 Faculty from 6 different institutions took part. The authors identified differences with higher ratings students/faculty and vice versa. One specific result for a possible improvement of the curriculum was identified in the area of pharmacology. Limitations of the study were presented in detail by the authors.A very interesting study for colleagues working in this area.

Reviewer Expertise:

NA

No decision status is available

# Reviewer report: Reviewer response for version 1

**DOI:** 10.21956/mep.19635.r29247

**Published:** 2018-09-18

**Authors:** BALAJI ARUMUGAM

**Affiliations:** 1 TAGORE MEDICAL COLLEGE AND HOSPITAL

This review has been migrated. The reviewer awarded 4 stars out of 5 I appreciate all the authors for the detailed quantitative analysis of testing of skill proficiency at the end of EM programs. Usually this type of studies will be done by before and after testing of the same skills proficiency among the students. This study differs in testing the students perceived skill proficiency and faculty expectation skill proficiency in multiple institutions. This study has examined learners at a different stage of training and assessed a different set of skills and confirmed that there is a significant difference between student self -assessed proficiency and faculty expectations.Its surprising to see the results where the students rate their proficiency higher than faculty expectations. Of course the author has quoted the possible reasons for the higher rating. But the I feel that the rating might also be higher because the subject variations among the faculty and the students. (the way of perceiving the skill proficiency questions)Nevertheless, the study was well designed and quantitatively analyzed, so that the curriculum development or changes focusing on the particular skills can be done easily based on student and faculty perceptions.

Reviewer Expertise:

NA

No decision status is available
